# Malignant Fibrous Histiocytoma of the Breast: A Case Report

**DOI:** 10.1155/2012/579245

**Published:** 2012-03-18

**Authors:** Dimosthenis Miliaras, Emmanouel Konstantinides

**Affiliations:** ^1^Laboratory of Histology and Embryology, Aristotle University of Thessaloniki School of Medicine, 54006 Thessaloniki, Greece; ^2^Department of Pathology, Geniki Kliniki, 54645 Thessaloniki, Greece; ^3^Department of Obstetrics and Gynaecology, Geniki Kliniki, 54645 Thessaloniki, Greece

## Abstract

This paper concerns a case of Malignant Fibrous Histiocytoma (MFH) of the Breast in a 73-years-old woman. A lump was found in her right breast, measuring 1.7 cm in diameter. Surgical excision followed, and pathology revealed a highly atypical spindle cell tumor. Immunohistochemistry along the histological features, excluded the possibility of carcinoma, malignant phyllodes tumor, or another type of pure sarcoma. There was no history of previous irradiation in the region. MFH is among the rarest types of sarcoma of the breast, and most of the times behaves aggressively. Excision with wide, free-of-tumor margins is the most effective treatment, when feasible.

## 1. Introduction

The most common malignant tumor of the breast is by far the invasive carcinoma of the ductal or lobular type. In contrast, sarcomas are rare and may be primary or metastatic, and pure or fibroepithelial, that is, malignant phyllodes tumor. The frequency of breast sarcomas in total is less than 1% of all the malignant tumors of the breast [1, 2]. Pure sarcomas could be angiosarcomas, osteosarcomas, liposarcomas, leiomyosarcomas, fibrosarcomas, rhabdomyosarcomas, dermatofibrosarcomas protuberans or malignant fibrous histiocytomas [3, 4]. Among them angiosarcoma is known to be the most frequent sarcoma type of the breast, while malignant fibrous histiocytoma (MFH) seems to be one of the rarer types. Breast sarcomas mostly appear in the fourth and fifth decade of life. Mean age is around 40 years old [4]. Gradually progressive swelling is the commonest presenting feature. Herein we describe the characteristics of a case with MFH of the breast with early detection.

## 2. Case Report

**Figure 1:**
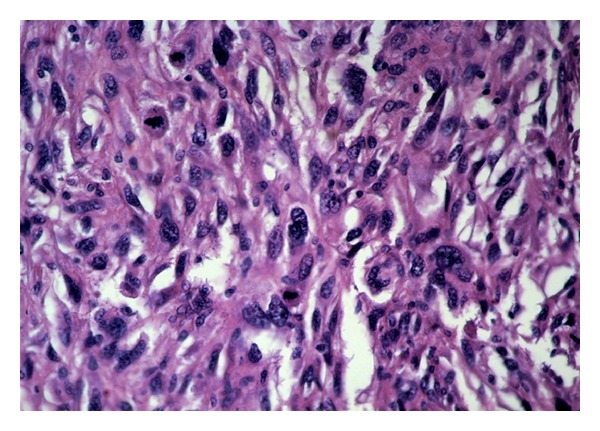
Highly atypical spindle cells arranged in fascicles, and presenting brisk mitotic activity (H&E, ×400).

**Figure 2:**
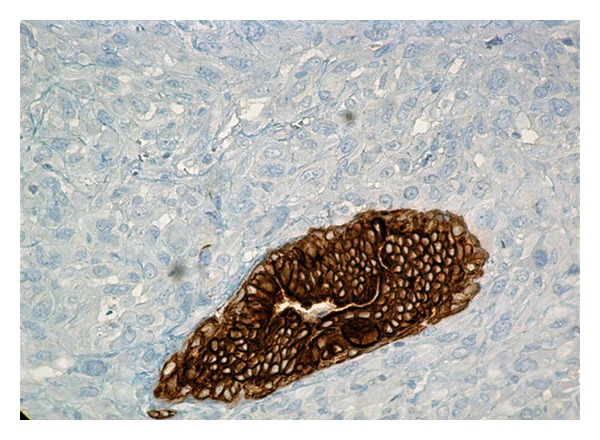
A remaining breast duct in the periphery of the tumor, immunoreactive to keratins AE1/AE3, surrounded by compactly arranged malignant cells, negative to keratins (DAB/Hematoxylin, ×400).

**Figure 3:**
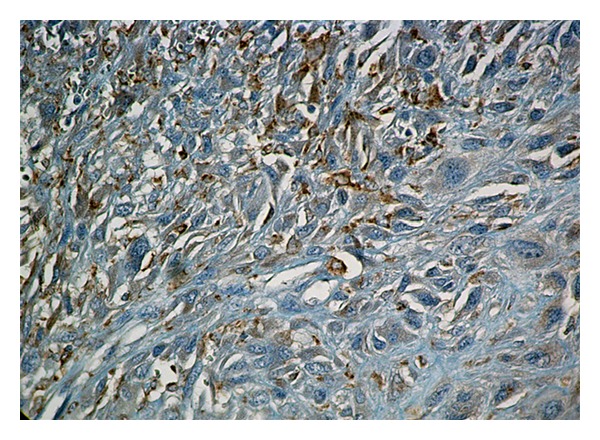
Many cancer cells are moderately to strongly positive to CD68 (DAB/Hematoxylin, ×400).

The present case concerned a white female, 73 years old. A lump was found by clinical examination in her right breast. On mammography the tumor was 1.7 cm in diameter and appeared as relatively circumscribed lesion. She was operated upon for this lump, and she had intraoperative frozen sections. The latter revealed a high-grade malignant tumor. A wide excision of the tumor along with ipsilateral axillary fat dissection followed. 25 Lymph nodes, 0.9–0.3 cm in diameter, were removed from the axillary fat. Paraffin sections showed a tumor composed of spindle cells, arranged in interlacing fascicles (Figure 1). Neoplastic cells presented pleomorphic nuclei, giant cell forms, and many and atypical mitoses. Malignant cells were infiltrating between pre-existing benign ducts in the periphery of the tumor (Figure 2). No intraepithelial lesion in the intratumor or the adjacent breast ducts was detected. Subsequently, immunohistochemistry was performed using an automated streptavidin-biotin method (Benchmark GX, Ventana Medical Systems, Tuscon, AZ, USA) in order to identify the origin of the tumor cells. The following antibodies were used: broad spectrum keratins (clone: AE1/AE3, dilution 1 : 50, Dako, Glostrup, Denmark), desmin (clone: es-der1, dilution 1 : 100, Newcastle upon Tyne, UK), a-smooth muscle actin (clone: asm1, dilution 1 : 50, Novocastra), CD34 (clone: Qbend, dilution 1 : 50, Dako), S-100 protein (clone: pol, dilution 1 : 400, Dako), CD68 (clone: KP-1, dilution 1 : 100, Dako), vimentin (clone: V9, dilution 1 : 100, Novocastra), estrogen receptor (clone: GF11, dilution 1 : 40, Novocastra), progesterone receptor (clone: 636, dilution 1 : 40, Dako), and HER2/neu (clone: CB11, dilution 1 : 100, Dako). Concurrently, for each antibody a positive and a negative control were carried out. Diaminobenzidine (DAB) was used as chromogen, and hematoxylin as counterstain. All of the above antibodies presented a negative reaction in the tumor cells, except CD68 (Figure 3) and vimentin. The surgical margins of the specimen were widely free of tumor. All removed axillary lymph nodes were also free of tumor. The histologic features described above, along with the immunohistochemical profile of the tumor rendered a diagnosis of malignant fibrous histiocytoma. No signs of distant metastases in the lungs or other organs were found in the postoperative investigation with the use of various imaging modalities (CT scan, MRI, Bone scan). Since the tumor was small (stage T1c, N0, M0), and widely excised, it was decided that no further treatment is necessary. Today the patient, 12 months after surgery, is alive and well with no evidence of recurrence of the disease.

## 3. Discussion

The most recent WHO fascicle on the classification of malignant tumors of the breast and female genital tract does not include MFH in the sarcoma types that could grow in the breast [5]. This exemplifies the rarity of this kind of tumor in the breast. Approximately 50 such cases have been reported in the literature thus far [1–4, 6–14]. This is contrast to other sites, and especially the soft tissues, where generally malignant fibrous histiocytoma is considered to be one of the most common sarcomas. Male breast may also develop malignant fibrous histiocytoma [15]. Many breast sarcomas, and especially angiosarcoma, arise frequently after radiotherapy [3]. Malignant fibrous histiocytomas may also develop in the breast after radiation therapy for breast carcinoma [13], while many reported cases seem to appear de novo [2, 7–12].

Among other spindle cell lesions of the breast, invasive carcinoma (i.e., metaplastic or sarcomatous carcinoma) is by far the most frequent and should be always considered before any other diagnosis, and excluded with the use of appropriate antibodies [14, 16]. Classically, MFH is composed by highly atypical spindle cells with giant and multinucleated cell forms, and many and atypical mitoses, arranged in fascicles or in a storiform pattern. Our case represents a genuine primary malignant fibrous histiocytoma of the breast. The possibility of metaplastic carcinoma was excluded by the negative reaction of the tumor cells to keratins AE1/AE3. Negativity to other antibodies, such as smooth muscle actin, desmin, S-100 protein, and CD34 excluded the event of another type of sarcoma such as leiomyosarcoma, liposarcoma, or angiosarcoma. In addition, the presence of exclusively sarcomatous elements within the tumor, without epithelial phylloid structures, along with the small size of the tumor established the diagnosis of a pure sarcoma, and not a fibroepithelial one (i.e., malignant phyllodes tumor). Our case is noteworthy, not only because of the infrequency of MFH in the breast, but also because of its relatively small size, and its de novo appearance, that is, without any previous medical treatment in this or any other site of the patient.

Breast sarcomas are aggressive tumors, even though there are only a few small series in the literature, and it is not easy to draw conclusions for their proper management. It could be thought that as superficially located sarcomas they might have a better prognosis than their deeper-seated counterparts. Still, Pandey et al. in series of 19 patients with breast sarcomas reported that the disease-free survival at 3 years was only 39% [4]. More recently, Hartel et al. in a series of 19 MFHs of the breast reported 33% overall mortality from the disease [15]. Local recurrence and distant metastases are common events. Sarcomas metastasize more frequently through blood vessels to the lungs, skin, liver, and bones. Lymph node metastases may also be found in some cases. However, it is debatable if axillary lymph node dissection should be a standard procedure in every case of breast sarcoma, considering the side effects of this operation. Wide excision with tumor-free margins is recommended, when feasible [15]. Radiation therapy and chemotherapy has also been used as adjunct therapeutic approaches in some cases [4]. Finally, we would like to stress the significance of early detection, as it happened in our case, which obviously has the greatest impact in the prognosis of MFH as in any other type of malignant tumor.
